# Influence of RAFT Agent on the Mechanism of Copolymerization of Polypropylene Glycol Maleinate with Acrylic Acid

**DOI:** 10.3390/polym14091884

**Published:** 2022-05-05

**Authors:** Meiram Zh. Burkeev, Meruyert S. Zhunissova, Yerkeblan M. Tazhbayev, Vitaliy N. Fomin, Akmaral Zh. Sarsenbekova, Gulsym K. Burkeyeva, Akerke T. Kazhmuratova, Tolkyn S. Zhumagalieva, Elmira Zh. Zhakupbekova, Tolkyn O. Khamitova

**Affiliations:** 1 Chemical Faculty, NJSC “Karaganda University Named after Academician E.A. Buketov”, Karaganda 100000, Kazakhstan; m_burkeev@mail.ru (M.Z.B.); tazhbaev@mail.ru (Y.M.T.); vitfomin@mail.ru (V.N.F.); chem_akmaral@mail.ru (A.Z.S.); guls_b@mail.ru (G.K.B.); kazhmuratova@mail.ru (A.T.K.); rektor@ksu.kz (T.S.Z.); elmira_zhakupbek@mail.ru (E.Z.Z.); 2 Department of Soil Science and Agrochemistry of the Agronomic, Faculty of the Kazakh Agrotechnical, University named after Saken Seifullin, Nur-Sultan 010000, Kazakhstan; khamitova.t@inbox.ru

**Keywords:** unsaturated polyester, polypropylene glycol maleate, acrylic acid RAFT polymerization

## Abstract

Studies have shown the possibility of synthesizing new polymers based on polypropylene glycol maleate with acrylic acid in the presence of a RAFT agent (2-Cyano-2-propyl dodecyl trithiocarbonate CPDT). The effect of RAFT agent concentration on network density has been shown to be connected with product yield. Herein, the composition of the obtained copolymers was determined using FTIR spectrometry in combination with the chemometric method of partial least squares (or projection to latent structures). To investigate the synthesized hydrogels, the degrees of equilibrium swelling was studied. The resulting objects were characterized by infrared spectroscopy. The surface morphology of the polymers was studied and the pore sizes were estimated using scanning electron microscopy. The structure of the test samples was confirmed by NMR spectroscopy. The thermal stability of crosslinked polymers was determined using thermogravimetry.

## 1. Introduction

At present, the chemical modification of polymers is of particular importance for improving their properties and significantly expanding the scope of their usage. [1]. In this regard, the development and study of new materials with controlled characteristics and polyfunctional properties is one of the key issues in modern polymer chemistry [2]. One of the advanced comonomers for the synthesis of these materials is unsaturated polyester resin.

Unsaturated polyester resins are a group of thermosetting polymers that are light yellow to dark brown viscous honey-like liquids with a reactive vinyl group [3].

The interaction of unsaturated polyesters with vinyl monomers is called the Benig copolymerization reaction [4]. Studies have reported results with regard to copolymerization with vinyl acetate, styrene, and methyl methacrylate [5], allyl ethers and complex ethers, and vinyl formiates [6].

Based on analysis of the literature and patent searches, there are few data on the curing of unsaturated polyesters with ionic comonomers, despite the fact that this process enables the synthesization of “intelligent” polymers. Accordingly, we obtained cross-linked copolymers based on polyethylene glycol maleinate with acrylic and methacrylic acids and acrylamide, which showed satisfactory moisture-absorbing properties [7].

When carrying out the copolymerization reaction of unsaturated polyester resins with a family of ionogenic monomers, there are obtained spatially cross-linked copolymers with a chaotic arrangement of the network in space. As practice shows, this is because radical polymerization does not fully allow the synthesis of polymers with the required properties [8].

In this regard, RAFT polymerization is of considerable interest, as it enables replacement of the uncontrolled chain termination reactions of a macromolecule with reversible reactions in the presence of RAFT agents, which then makes it possible to control quadratic chain termination [9,10]. Reactions between reactive macroradicals with a chain transfer agent enable the formation of a new potential center for subsequent chain growth. Thus, the polymerizing system contains both growing macroradicals and “dormant” macromolecules, which at a certain moment can split off the radical and continue the growth of the polymer chain, which together gives rise to the so-called “pseudo-living” polymerization process [11,12].

The use of such approaches in the copolymerization of unsaturated polyester resins with ionogenic monomers opens up unlimited possibilities for controlling the size of the three-dimensional network. It facilitates the design of a macromolecule in the composition of a polymer with a known molecular weight, block, and graft copolymers [13].

Previously, we carried out preliminary studies of the reactions of copolymerization of polypropylene glycol maleinate (*p*-PGM) with acrylic acid (AA) in the presence of a RAFT agent [14]. It has been established that the presence of a RAFT agent affects both the density of the polymer network and the formation of a branched polymer. The obtained results showed that the synthesized crosslinked copolymers have high sorbing properties and indicate the possibility of their use as smart systems, as well as efficient nanocatalysts [15]. In this work, it seemed interesting to investigate in detail the direction of the copolymerization reaction of *p*-PGM with AA depending on the concentration of the RAFT agent.

## 2. Experimental Section

The following reagents were used in the work: propylene glycol (Sigma-Aldrich, Burlington, MA, USA), maleic anhydride (Sigma-Aldrich), acrylic acid (AA) (Sigma-Aldrich), benzoyl peroxide (BP) (Sigma-Aldrich), zinc chloride (Sigma-Aldrich), 1,4-dioxane (Sigma-Aldrich), and RAFT agent (2-Cyano-2-propyl dodecyl trithiocarbonate CPDT) from Sigma-Aldrich.

The original polypropylene glycol maleate (*p*-PGM) was obtained by polycondensation of maleic anhydride with propylene glycol alcohol at a temperature of 423–453 K, according to the standard procedure [16]. The molecular weight of *p*-PGM was determined using gel permeation chromatography (GPC).

Radical copolymerization of *p*-PGM with AA (50:50 mol.%) was carried out at a temperature of 343 K in organic solvent dioxane (by weight 1:1) in the presence of benzoyl peroxide (BP) as an initiator. The ampoules with the mixture were degassed for 30 min, then the copolymerization was kept for 52 h. The synthesized polymers were washed several times with dioxane and dried at a temperature of 323–333 K until a constant mass was established in a vacuum drying cabinet.

RAFT polymerization of *p*-PGM with AA was carried out under the same conditions as in radical copolymerization, but with the addition of different concentrations of the RAFT agent. Ampoules with solutions were purged with an inert gas in a vacuum unit and closed. Subsequently, the ampoules were kept in a thermostat where the temperature was maintained at 343 K with an accuracy of ±0.10 °C for 52 h. Upon completion of the copolymerization, the ampoules were cooled and opened. The synthesized products were quantitatively separated; the cross-linked polymers were then filtered from the mother liquor and dried in a vacuum drying cabinet at a temperature of 323–333 K to a stable mass. Further, branched copolymers were obtained by the precipitation in alcohol of the mother liquor [17].

The ^1^H and ^13^C NMR spectra of the resulting branched copolymers *p*-PGM:AA:CPDT and the starting *p*-PGM oligomer were recorded on JNM-ECA Jeol 400 spectrometer (frequency 399.78 and 100.53 MHz, respectively) using a CDCl_3_ solvent. Chemical shifts were measured relative to the signals of residual protons or carbon atoms of deuterated chloroform.

The composition of the obtained products was determined by HPLC by the number of unreacted monomers on a Shimadzu (Kyoto, Japan) chromatograph, as well as by IR spectrometry.

The synthesized polymers were homogenized by grinding with potassium bromide in a mechanical mortar and pressed into tablets, which were used to record IR spectra. The instrument resolution was 1 cm^−1^, and each spectrum was averaged over 50 scans.

The surface topography of the synthesized objects was performed using a MIRA3 scanning electron microscope (SEM) (Tescan, Brno, the Czech Republic).

The swelling behavior of the copolymers was determined by the gravimetric method according to the formula: [17]

$$\alpha \;\left(\%\right)=\frac{m_{1}-m_{0}}{m_{0}}\times 100$$

where *m*_1_ and *m*_0_ represent masses of swollen polymer and dry polymer, respectively.

## 3. Results and Discussion

It is known that the products of copolymerization of unsaturated polyester resins with vinyl monomers are refractory and cross-linked copolymers [7]. In addition, our previous studies showed copolymerization with ionogenic monomers, which also leads to the formation of cross-linked copolymers at any ratios of the initial monomers. In this work, we studied the copolymerization of *p*-PGM with AA with the addition of a RAFT agent. The calculated copolymerization data are presented in Table 1.

**Figure 1:**
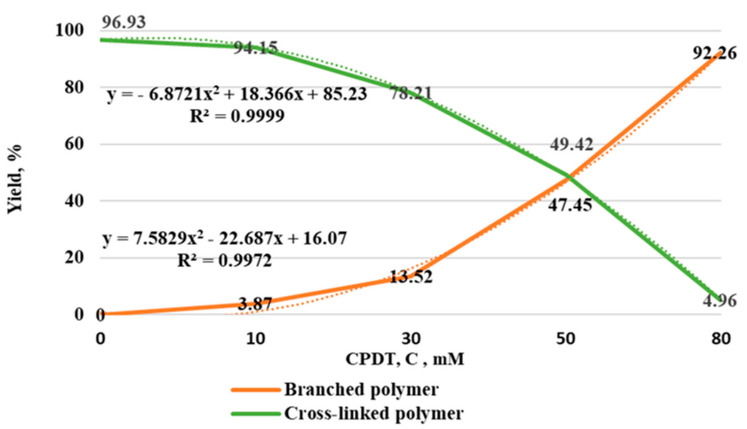
Dependence of the concentration of the RAFT-agent on the yield of copolymers.

Table 1 shows that both soluble branched products and cross-linked copolymers are formed as a result of copolymerization. It was found that the greater the concentration of the RAFT agent, the greater the yield of soluble products. In addition, an increase in the concentration of the RAFT agent leads to an increase in the swelling capacity of the copolymers, which is associated with a decrease in the degree of crosslinking. Graphically, the results of the study are presented in Figure 1.

The graph (Figure 1) illustrates that the yield dependence of the branched polymer increased in proportion to the square of the concentration of the RAFT agent. Correspondingly, the yield of the cross-linked copolymer decreased in proportion to the square of its concentration [18].

FTIR spectrometry was used in combination with the chemometric method of partial least squares (or projection to latent structures) [19], implemented in the R environment [20,21] to determine the mass fraction of *p*-AA and *p*-PGM units in the obtained polymer samples. The mixtures of *p*-AA and *p*-PGM homopolymers containing 1.0, 3.0, 5.0, 7.0, 9.0, 10.0% *p*-AA, and *p*-PGM up to 100% were prepared for calibration by the method of long-term joint attrition of the components with potassium chloride in a mechanized agate mortar within 30 min. The attrition time was chosen in such a way as to achieve the highest possible reproducibility of the IR spectrum in three parallel samples. The sample preparation of the studied polymer samples was carried out similarly. Three tablets containing 4.0 mg of polymers in 300.0 mg of KBr were pressed from the homogenized mixture, and their infrared spectra were recorded in the range of 450 4000 cm^−1^. Numerical data from the spectra were imported into the R environment for further mathematical processing. The noisiest part of the spectrum 3700–4000 cm^−1^ was excluded from processing. Using standard samples, the model was trained with cross-validation. R^2^ comprised 0.998. Based on the spectra of the samples, the mass fraction of *p*-AA units in the copolymers was calculated (Table 1), taking into account the Student’s coefficient for three repetitions at a confidence level of *p* = 0.95. Mass values were then converted to molar values.

The general view of the IR spectra of cross-linked and branched copolymers was previously published by us [14].

**Figure 2:**
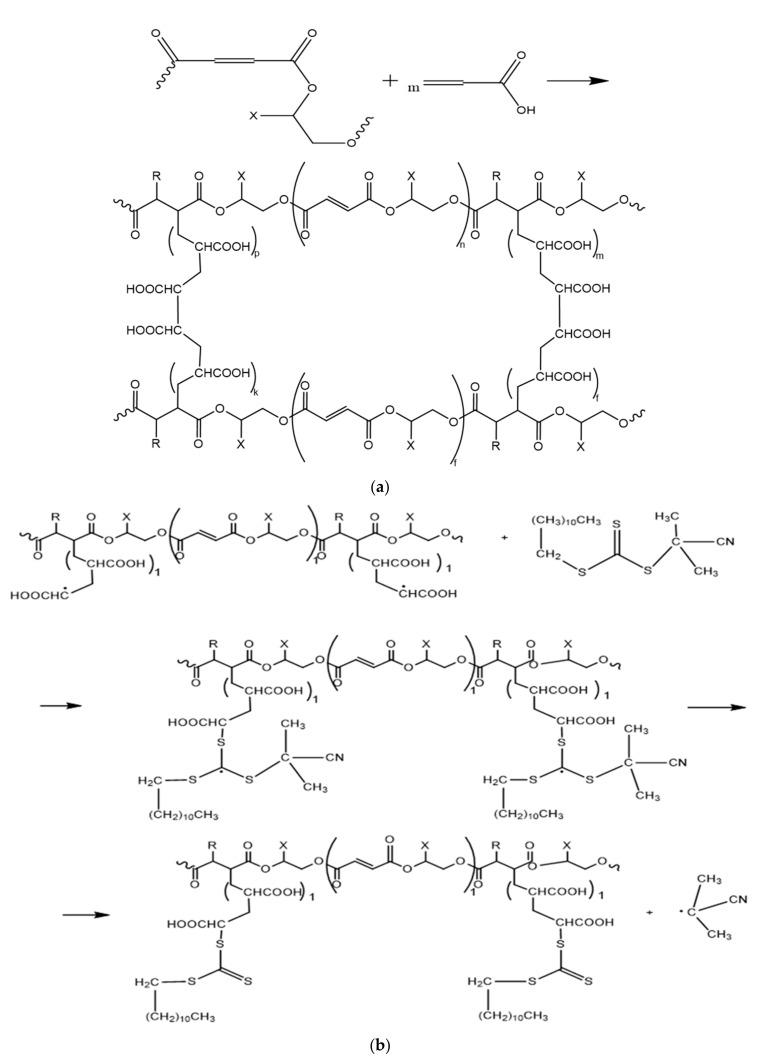
Schematic structure of *p*-PGM:AA (**a**), *p*-PGM:AA:CPDT (**b**).

The mechanism of the copolymerization reaction of *p*-PGM:AA (a) and *p*-PGM:AA:CPDT (b) in the presence of a benzoyl peroxide initiator can proceed according to the scheme shown in Figure 2:

It follows from the scheme shown in Figure 2b that the addition of a RAFT agent reduces the proportion of bimolecular chain terminations, leading to the formation of cross-linked copolymers and an increase in the yield of branched copolymers.

**Figure 3:**
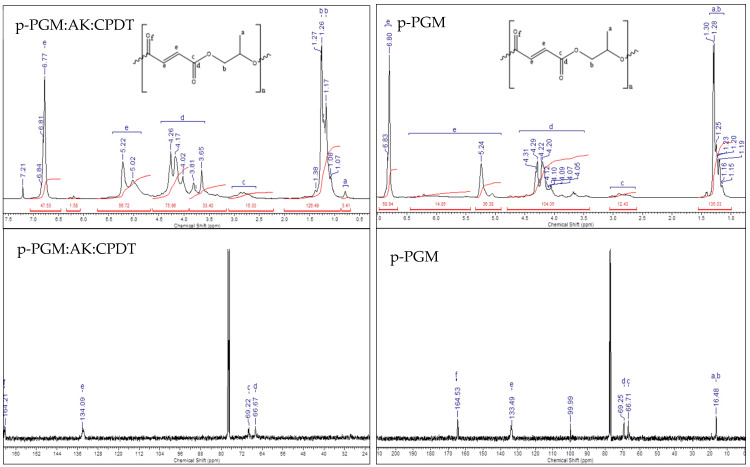
IR spectra of copolymers of *p*-PGM:AA:[CPDT] = 80 mM.

In continuation of the work, the data of NMR spectra of branched copolymers were obtained, confirming the presence of maleic groups and groups characteristic of polypropylene glycol maleate, which corresponded to the data of the initial oligomer (Figure 3). The ^1^H NMR spectrum of the branched copolymer *p*-PGM:AA:CPDT exhibited a small singlet signal at 0.79 ppm, which is characteristic of the protons of the methyl group of Ha. The intense multiplet signal in the region of 1.07–1.60 ppm. corresponded to the protons of the Hb methylene groups, which were adjacent to the oxygen atoms in the polymer chain. Two multiplet signals at 2.54–2.99 and 3.34–4.44 ppm corresponded to the protons of simple and ester methine and methylene groups of the aliphatic series of Hc and Hd. Multiplet signals of medium intensity at 5.02–5.22 and a high-intensity multiplet at 6.77–6.81 ppm. indicated the presence of a large amount of He ethylene protons in the copolymer. The location of ethylene protons in two different multiplets is associated with their spatial orientation—cis-, and trans-, as well as the influence of neighboring functional groups on ethylene protons. Ethylene protons located in the multiplet at 6.77–6.81 ppm were characterized by the presence of a larger number of electronegative atoms—oxygen atoms—than protons recorded at 5.02–5.22 ppm.

In the ^13^C NMR spectrum of the copolymer, methyl and methylene carbon atoms Ca and Cb were detected at 16.33 ppm. The carbon atoms of the alkyl fragment adjacent to and associated with simple and complex ether groups Cc and Cd were observed at 66.87 and 69.40 ppm. Ethylene carbon atoms Ce appeared at 133.83 ppm. The carbon atoms of carboxyl groups and ester fragments appeared at 164.28 ppm.

To determine the surface morphology of the polymers, an SEM with a high-performance silicon drift detector X-Act was used at an accelerating voltage of 5 kV. The BSE and SE detectors were used to obtain images with a magnification of 20.1 kx. The BSE detector showed the material (compositional) contrast, with the field of view at 13.2 nm and the pixel extension at 1024–1024 [22].

**Figure 4:**
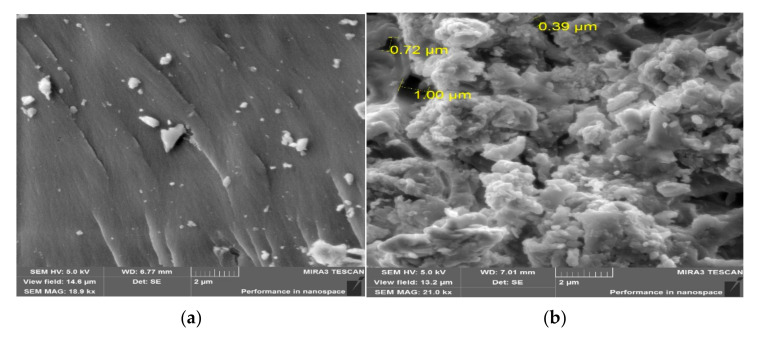
Topography of the polymer *p*-PGM:AA (**a**); surface morphology of the polymer *p*-PGM:AA:[CPDT] = 80 mM (**b**).

On the micrograph (Figure 4), it was observed that the surfaces of the particles of *p*-PGM:AA samples (a) had hard and brittle cleavage sites.

In the course of microanalysis, the *p*-PGM:AA:CPDT copolymers (b) showed signs of a loose surface and the formation of a layered and porous structure in comparison with the *p*-PGM:AA copolymers. In addition, the analysis revealed pores. It is evident from the SEM topography that an increase in the concentration of the RAFT agent leads to the formation of a network and affects the sizes of the formed pores, which give a high swelling capacity to the polymers.

**Figure 5:**
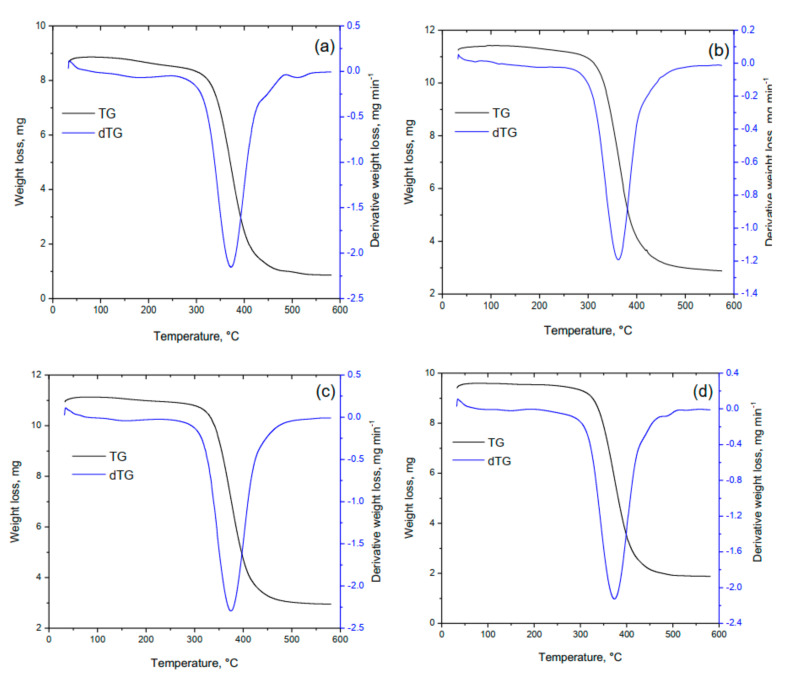
TG curves of analysis of *p*-PGM:AA copolymer (**a**) and *p*-PGM:AA:CPDT copolymers obtained in the presence of 10 mM (**b**), 30 mM (**c**), 50 mM (**d**) RAFT-agent.

In continuation of the study of the physicochemical properties of polymers, a thermogravimetric analysis of the samples was carried out. To study the thermal behavior of the copolymers, *p*-PGM:AA and *p*-PGM:AA:CPDT samples were used. Thermogravimetric curves (TG), the results of the analysis of the decomposition of copolymers (weight loss (∆m/m_0_, mg), and the rate of weight loss from temperature ((dm/dt)/m_0_, mg min^−1^) according to the data obtained are presented in Figure 5 and Table 2.

As can be seen, the thermograms (Figure 5) have a similar character with the onset of thermal degradation at 320 °C, indicating a similar mechanism in the behavior of polymers during degradation. It was found that for branched and spatially cross-linked copolymers, the thermogravimetric analysis showed the presence of single decomposition peaks (Figure 5, dTG curves). As can be seen from the dTG curve (Table 2, Figure 5a), the process of thermal degradation of the spatially cross-linked copolymer occurred at a temperature of ~3100~4000 °C, while the total weight loss of the sample was ~6.9 mg of the initial sample weight. The most intensive decomposition of the branched copolymer *p*-PGM:AA with the RAFT agent, associated with the splitting of the main macromolecular chain (accompanied by the release of gaseous products) and the final destruction of the polymer, took place in the temperature range of ~320.00 °C~420.00 °C, as evidenced by the characteristic peaks at dTG curves (Table 2, Figure 5b) with a maximum Tmax. = 372.0 ± 0.10 °C at a heating rate of 10.00 °C/min. The weight loss due to thermal degradation of the branched copolymer obtained in the presence of the RAFT agent ([CPDT] = 50 mM) was lower, and the temperature of the maximum rate of its thermal decomposition was higher than for the cross-linked copolymer (Table 2). This indicates that the mass fractions of the branched copolymers (prepared in the presence of the RAFT reagent at concentrations of 10 mM, 30 mM, and 50 mM) were higher than those of the cross-linked copolymer, and their thermal decomposition occurred at higher temperatures.

**Figure 6:**
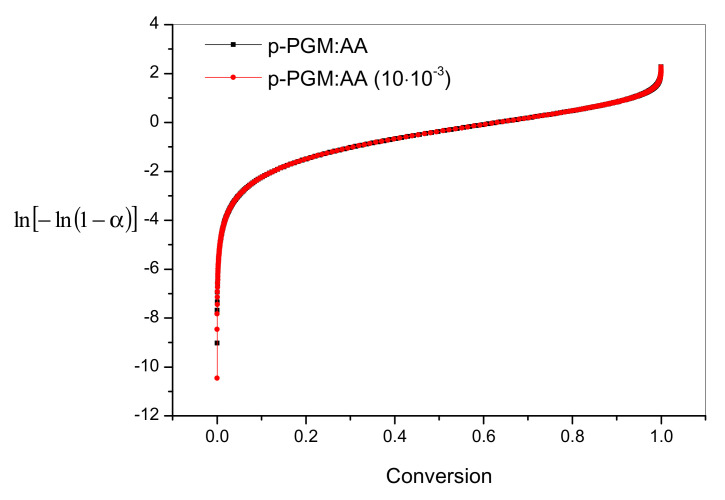
Family of curves f(α) from the degree of conversion (α) for copolymers *p*-PGM:AA and *p*-PGM:AA:CPDT (10 mM).

To compare the thermal stability of the *p*-PGM:AA and *p*-PGM:AA:CPDT copolymers, apparent activation energies of the decomposition reaction were determined. Comparing the calculations of thermal degradation of the *p*-PGM:AA and *p*-PGM:AA:CPDT copolymers, it can be argued that the apparent activation energies of the *p*-PGM:AA and *p*-PGM:AA:CPDT copolymers ([CPDT] = 10 mM) were different at the same transformation temperature (α). The use of non-isothermal methods enabled the obtainment of the dependence of Ea on the degree of conversion (α). Experimental data are presented in the form of graphs showing the dependence of f(α) on α. Figure 6 shows a family of curves obtained at one heating rate (β = 10 °C/min) for *p*-PGM:AA and *p*-PGM:AA:[CPDT] = 10 mM. Each value of α corresponds to several values of the velocity f(α). Thus, for any value of α, one can construct the dependence of lnf(α) on 1/T and calculate the value of apparent activation energy for a given α (Table 3).

It emerged that the *p*-PGM:AA:CPDT copolymers were characterized by a significantly higher thermal stability (Ea = 171.38 kJ mol^−1^) compared to the *p*-PGM:AA copolymer (E_a_ = 124.52 kJ mol^−1^) obtained by traditional radical copolymerization. Therefore, it can be concluded that *p*-PGM:AA:CPDT copolymers obtained by RAFT(co)polymerization require more energy to break the strong bond in the polymer chain. It should be noted that the thermal stability of copolymers obtained by RAFT (co)polymerization increased in the following order: *p*-PGM:AA:[CPDT] = 10 mM < *p*-PGM:AA:[CPDT] = 30 mM < *p*-PGM:AK:[CPDT] = 50 mM. Thus, generalizing the experimental data on the study of thermal stability, we are convinced that RAFT copolymers have a relatively high degree of resistance to heating, which is apparently due to a decrease in the concentration of acrylic acid residues in the side branches (Figure 2).

## 4. Conclusions

Using the copolymerization reactions of *p*-PGM with AA, it was found that the addition of small amounts of a chain transfer agent changed the direction of the reactions with the formation of spatially cross-linked and branched copolymers.

Based on the SEM data, it was found that the samples containing the chain transfer agent had a looser, more porous structure with an average pore size of between 0.39 nm and 1.00 nm.

Thermogravimetric analysis methods showed an increase in the thermal stability of cross-linked copolymers with an increase in the concentration of the RAFT agent.

The weight method for determining the swelling capacity of the obtained polymers proved that the greater the amount of CPDT in the composition of the initial polymer-monomer mixture, the greater the sorption capacity of the obtained products.

Thus, establishing the dependence of the density and number of the network on the concentration of the RAFT agent makes it possible to “regulate” the construction of the resulting copolymers, resulting in more predictable structures. By changing the concentration of the chain transfer agent, it is possible to obtain polymers with a controlled degree of crosslinking and swelling ability.

## Figures and Tables

**Table 1:** Radical copolymerization of *p*-PGM with AA in a dioxane solution in the presence of a RAFT agent. [M_1_]:[M_2_] = 49.9:50.1 mol.%, T = 343 K, [I] = 8 mM.

[RAFT], mM	Cross-Linked Copolymer			Branched Copolymer	
	Yield, %	[m_1_]:[m_2_] Mol.%	Swelling, α, %	Yield, %	[m_1_]:[m_2_] Mol.%
-	96.93	48.21:51.79	187.12	-	-
10.02	94.15	45.45:54.55	201.16	3.87	31.58:68.42
30.01	78.21	46.15:53.85	267.21	13.52	33.33:66.67
50.01	49.42	47.06:52.94	314.17	47.45	43.75:56.25
80.03	4.96	47.06:52.94	385.11	92.26	45.45:54.55

**Table 2:** Results of TG analysis of the decomposition of *p*-PGM:AA and *p*-PGM:AA:CPDT copolymers in a nitrogen atmosphere.

[RAFT], mM	T_i_, °C	T_f_, °C	T_max_, °C	Weight Loss, mg
-	310.0	400.0	362.0	6.9
10.02	325.0	420.0	372.0	7.4
30.01	330.0	423.0	372.0	7.2
50.01	326.0	420.0	374.0	6.7

**Table 3:** Values of apparent activation energy of *p*-PGM:AA and *p*-PGM:AA:CPDT copolymers in a nitrogen atmosphere.

[RAFT], mM	E_a_, kJ/Mol	A, s^−1^
-	124.52	6.29 × 10^12^
10.02	126.27	1.60 × 10^13^
30.01	146.23	4.35 × 10^14^
50.01	171.38	8.86 × 10^16^

## Data Availability

All data presented in this paper are available upon reguest from the corresponding author.
